# Deployment of an Artificial Intelligence Histology Tool to Aid Qualitative Assessment of Histopathology Using the Nancy Histopathology Index in Ulcerative Colitis

**DOI:** 10.1093/ibd/izae204

**Published:** 2024-09-16

**Authors:** David T Rubin, Olga Kubassova, Christopher R Weber, Shashi Adsul, Marcelo Freire, Luc Biedermann, Viktor H Koelzer, Brian Bressler, Wei Xiong, Jan H Niess, Matthias S Matter, Uri Kopylov, Iris Barshack, Chen Mayer, Fernando Magro, Fatima Carneiro, Nitsan Maharshak, Ariel Greenberg, Simon Hart, Jamshid Dehmeshki, Laurent Peyrin-Biroulet

**Affiliations:** Department of Pathology, University of Chicago, Chicago, IL, USA; Image Analysis Group, London, UK; Inflammatory Bowel Disease Center, University of Chicago Medicine, Chicago, IL, USA; Takeda, Cambridge, MA, USA; Takeda, Cambridge, MA, USA; University Hospital of Zurich, University of Zurich, Zurich, Switzerland; Department of Pathology and Molecular Pathology, University Hospital and University of Zurich, Zurich, Switzerland; Institute of Medical Genetics and Pathology, University Hospital Basel, Basel, Switzerland; Faculty of Medicine, University of British Columbia, Vancouver, BC, Canada; Faculty of Medicine, University of British Columbia, Vancouver, BC, Canada; Department of Biomedicine and University Digestive Healthcare Center, University of Basel, Clarunis, Basel, Switzerland; Institute of Medical Genetics and Pathology, University Hospital Basel, Basel, Switzerland; Chaim Sheba Medical Center Ramat Gan Israel, Ramat Gan, Israel; Chaim Sheba Medical Center Ramat Gan Israel, Ramat Gan, Israel; Chaim Sheba Medical Center Ramat Gan Israel, Ramat Gan, Israel; Center for Health Technology and Services Research (CINTESIS@RISE), Faculty of Medicine, University of Porto, Porto, Portugal; Faculty of Medicine, University of Porto and ULS São João, Porto, Portugal; Tel Aviv Sourasky Medical Center affiliated with the Faculty of Medical and Health Sciences, Tel Aviv University, Tel Aviv, Israel; Tel Aviv Sourasky Medical Center affiliated with the Faculty of Medical and Health Sciences, Tel Aviv University, Tel Aviv, Israel; Image Analysis Group, London, UK; Image Analysis Group, London, UK; Faculty of Engineering, Computing and the Environment, Kingston University London, London, UK; Department of Gastroenterology, INFINY Institute, INSERM NGERE, CHRU Nancy, F-54500 Vandoeuvre-lès-Nancy, France

**Keywords:** ulcerative colitis, artificial intelligence, histopathology

## Abstract

**Background:**

Ulcerative colitis (UC) is a chronic inflammatory bowel disease characterized by increased stool frequency, rectal bleeding, and urgency. To streamline the quantitative assessment of histopathology using the Nancy Index in UC patients, we developed a novel artificial intelligence (AI) tool based on deep learning and tested it in a proof-of-concept trial. In this study, we report the performance of a modified version of the AI tool.

**Methods:**

Nine sites from 6 countries were included. Patients were aged ≥18 years and had UC. Slides were prepared with hematoxylin and eosin staining. A total of 791 images were divided into 2 groups: 630 for training the tool and 161 for testing vs expert histopathologist assessment. The refined AI histology tool utilized a 4-neural network structure to characterize images into a series of cell and tissue type combinations and locations, and then 1 classifier module assigned a Nancy Index score.

**Results:**

In comparison with the proof-of-concept tool, each feature demonstrated an improvement in accuracy. Confusion matrix analysis demonstrated an 80% correlation between predicted and true labels for Nancy scores of 0 or 4; a 96% correlation for a true score of 0 being predicted as 0 or 1; and a 100% correlation for a true score of 2 being predicted as 2 or 3. The Nancy metric (which evaluated Nancy Index prediction) was 74.9% compared with 72.3% for the proof-of-concept model.

**Conclusions:**

We have developed a modified AI histology tool in UC that correlates highly with histopathologists’ assessments and suggests promising potential for its clinical application.

**Key Messages**

**What is already known?**

Histological remission is an aspirational target for ulcerative colitis treatment; however, scoring of histological images is time-consuming and prone to inter- and intraobserver variability.

**What is new here?**

Using an expanded population-diversified training dataset from 9 global study sites, we describe the accuracy and robustness of an AI tool for classification of histology images from ulcerative colitis patients using the Nancy Index.

**How can this study help patient care?**

Further development of this AI histology tool has potential to improve many elements of histopathological assessment of disease activity to aid treatment decisions.

## Introduction

Ulcerative colitis (UC) is a chronic relapsing and remitting inflammatory bowel disease (IBD), characterized by mucosal inflammation that usually starts distally in the rectum and can extend proximally to involve the length of the colon.^1^ Therapy for UC aims to provide rapid relief of clinical symptoms to maintain health-related quality of life and avoid disability by achieving endoscopic healing where possible, which is associated with improved long-term outcomes.^2,3^ Histological remission (variously defined) is currently considered to be an aspirational therapeutic target for the prevention of long-term complications and disease relapse upon both continuation and de-escalation of established maintenance therapy.^3^ Histological remission is also superior to endoscopic mucosal healing in predicting rates of subsequent neoplastic transformation of the affected colorectal epithelium.^4,5^

Although histological remission is included as a secondary outcome in UC clinical trials, there are a number of barriers to its widespread integration into clinical practice.^6^ These barriers include the large number (>30) of histological scoring indices available and the difficult and time-consuming nature of grading histological severity. Scoring histological remission requires dedicated training, is not considered standard practice by histopathologists in clinical practice, and is limited by high interobserver variability when it is used in clinical trials.^7^ In addition, the desirable inclusion of centrally read histological disease activity as an additional end point in clinical trials is associated with considerable logistical complexity, including the shipment of biomaterial across international borders.

The availability of digitized medical data from patients with IBD, including pathological imaging and histology data together with computational methods for complex pattern recognition and data analytics, has resulted in the emergence of the use of artificial intelligence (AI) in IBD.^8^ The aim of this project was to develop a novel AI histology tool based on a deep learning approach using histopathology slides from patients with UC.

## Methods

This multiphase project involved the initial development of the AI histology tool^9^ and a single-center proof-of-concept study (proof-of-concept phase) followed by a multicenter expansion to enhance the performance of the AI histology tool (rollout phase). Here, we describe the results from the rollout phase and describe how the AI histology tool has been refined and modified to analyze a broader range of cell characteristics.

### Dataset

Nine sites from 6 countries (France, *n* = 1; Canada, *n* = 1; Switzerland, *n* = 3; Israel, *n* = 2; Portugal, *n* = 1; the United States, *n* = 1) were included in the study. Patients were aged 18 years or older and had UC. Slides were prepared with hematoxylin and eosin staining and were scanned to produce digitized images. The images were digitized by the study sites at 20× magnification and 32-bit color, with a resolution between 1200 × 900 pixels and 2500 × 2500 pixels. The image files were then pseudo-anonymized and transmitted to the central, secure, cloud-based storage location managed by Image Analysis Group (IAG, London, UK). Images were excluded by the site if they were distorted or “noisy” with excessive slide sectioning artifacts. In addition, images underwent quality control by IAG to ensure their usability for the AI histology tool.

Each site contributed up to 40 digitized slides from patients with UC, distributed approximately evenly by disease stage, disease severity, sex, and age, to ensure an unbiased dataset for training and testing the AI histology tool. Whole slide images (WSIs) were collected from sites. Images were generated from the WSIs by dividing the large WSIs into smaller fields. A further 300 slides with similarly distributed features, from patients with UC, were additionally sourced by IAG. These images were added to the existing AI histology tool dataset of 200 slides, used in the proof-of-concept phase of the project.

Four centrally located expert IBD pathologists annotated the images, assigning a Nancy score to each image and identifying architectural features including epithelium and crypts, and cell types including eosinophils and neutrophils. Each image was scored according to the Nancy Index by at least 2 pathologists, and the consensus score was considered final. Architectural cellular features in the image were marked by one of the pathologists and verified by one other in the reader group.

After quality control, a total of 791 images from 791 separate patients were available for use in the study. The 791-image dataset was divided into 2 groups: 80% (630 images) were used for training and 20% (161 images completely new to the AI tool) were used for testing the AI histology tool.

### AI Histology Tool

**Figure 1.**
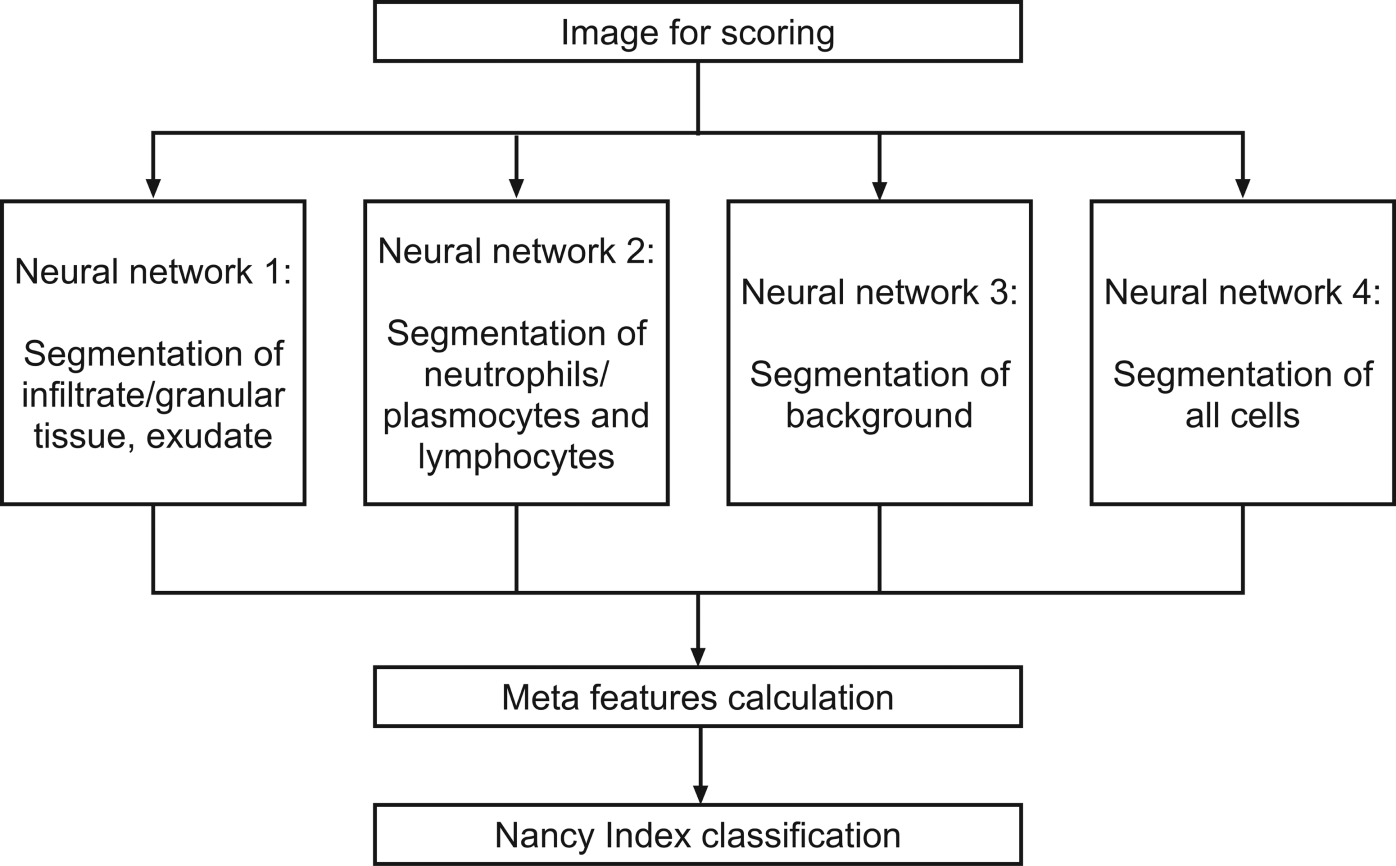
A schematic representation of the artificial intelligence tool workflow.

The AI histology tool utilized a 4-neural network structure to characterize new images into cell and tissue type combinations and locations. Subsequently, a classifier module assigned a Nancy Index score to each image. This was a refinement/modification of the proof-of-concept model, which used a 3-neural network structure to characterize images into cell and tissue type combinations and locations before the classifier module assigned a Nancy Index score.^9^ The 4 segmentation neural networks were each configured to detect different combinations of cells, cell density, and tissue types, known as meta-features (Figure 1). Based on the meta-feature output from the 4 segmentation networks, the classifier module predicted the Nancy Index score for the image using a random forest classification model (Figure 1).

The meta-features included in the model were neutrophils within the biopsy tissue area (not in the background; defined as blank areas without any tissue); granular tissue, exudate, and detritus within the biopsy tissue area (not in the background); and plasma cells and lymphocytes within the biopsy tissue area (not in the background).

The meta-feature output from each neural network was measured against a ground truth from the human pathologists reading the same image for the characteristics segmented by each network. An example of segmentation is provided in Supplementary Figure S1.

### AI Histology Tool Performance Assessment

As in the independent validation sample set, 75 new images not previously used in training were used to assess the AI histology tool’s histological performance. The AI histology tool was assessed in 3 parts: the segmentation matrix, the Nancy metric (which evaluated the Nancy Index prediction for each disease stage), and scoring prediction metrics. The performance of the rollout phase AI model was compared against the performance of the proof-of-concept model, which had been trained on only 200 images. These 200 images were also included in the training dataset for the rollout phase.^9^

#### Segmentation matrix

The performance of the 4 segmentation neural networks was assessed using the mean Intersection over Union (IoU). The IoU is the area of overlap between the AI-driven segmentation and the ground truth established by the histopathologist readers during the training and validation process, divided by the area of union between the AI segmentation and the ground truth. The IoU ranges from 0 to 1 (0% to 100%), with 0 signifying no overlap and 1 signifying perfectly overlapping segmentation. The mean IoU of the image was calculated as the average of each IoU for the 4 segmentation neural networks.

#### Nancy metric

The accuracy of the performance of the classifier module, which predicts the Nancy Index, was assessed using the fraction of predictions that the model assessed correctly (ie, the number of correct predictions over the total number of predictions made). Accuracy was represented as a step function, where the case was equal to 1 when the prediction was correct and equal to 0 when the prediction was incorrect. The function was smoothed and based on an asymmetric distribution. The argument of the function was the difference between the true Nancy Index value (the Nancy Index score assigned by the histopathologist) and the predicted Nancy Index value (the Nancy Index score assigned by the AI histology tool).

The Nancy Index prediction results were presented using a confusion matrix. A confusion matrix is a table that is often used to describe the performance of a classification model (or “classifier”) on a set of test data for which the true values are known. It allows the visualization of the performance of an algorithm. Each row of the matrix represents the instances in a predicted class, and each column represents the instances in an actual class (or vice versa).

##### Equation for the Nancy metric


$$\begin{array}{l} Nancy\;metric=\;\frac{\overset{N}{\underset{j=1}{\sum }}w_{j}^{i_{true}}*f_{j}\left(i_{true}-i_{pred}\right)}{\overset{N}{\underset{j=1}{\sum }}w_{j}^{i_{true}}}, \end{array}$$

where *f* = Nancy function, *i*_*true*_ = true class value, *i*_*pred*_ = predicated class value, *j* = sequential number of the object in the set, *w* = weight (importance), and *N* = number of objects in the set.

#### Scoring prediction metrics

If a prediction is correct, the function value is 1. If the prediction is wrong, the function value tends to 0, in proportion to the increase in the absolute value of the difference between the predicted and true Nancy Index values. The insensitivity of this tendency depends on the direction of difference. The value of the Nancy metric was determined by summing up the value of the function for all objects in the test set and calculating the average. The different importance of the Nancy Index was also considered.

#### Intraclass correlation

The intraclass correlation coefficient (ICC) was also reported. The ICC is a descriptive statistic that can be used when quantitative measurements are made on units that are organized into groups. The ICC describes the strength of resemblance between units in the same group.

## Results

Out of the 791 slides submitted by the 8 sites, histopathologists meticulously annotated 43 342 characterizations. These annotations encompass various elements such as neutrophils, plasmocytes, lymphocytes, eosinophils, stroma (lamina propria), granulation tissue, fibrinoleukocytic exudate, and vessels (Table 1). The numbers of slides for each grade were 102 (Grade 0), 105 (Grade 1), 114 (Grade 2), 283 (Grade 3), and 121 (Grade 4). In addition, there were 66 slides that had different scores from at least 3 histopathologists; these were undefined and excluded from the training set.

**Table 1.** Key characteristics relevant to ulcerative colitis annotated by histopathologists on digitized slide images.

Characterizations annotated	Count in total slides
Neutrophils	11 683
Plasmocytes	15 740
Lymphocytes	10 886
Eosinophils	3110
Stroma (lamina propria)	395
Granulation tissue	82
Fibrinoleukocytic exudate	84
Vessels	1362
Total characterizations	43 342

Mean IoU values for each cell type are presented in Table 2. The confusion matrix analysis for the proof-of-concept AI model and the pilot AI model is presented in Table 3. In comparison with the proof-of-concept AI tool, each segmented feature class demonstrated an improvement in accuracy in the rollout phase AI model, with an average accuracy of 67% for the rollout AI model compared with 63% for the proof-of-concept AI model. A confusion matrix analysis demonstrated an 80% correlation between predicted and true labels for Nancy scores of 0 or 4 (Table 3). The correlation was 96% for a true Nancy score of 0 being predicted by the AI tool as 0 or 1, and 100% for a true Nancy score of 2 being predicted by the AI tool as 2 or 3. The Nancy metric was 74.9% for the pilot AI model compared with 72.3% for the proof-of-concept model.

**Table 2.** Performance of the segmentation neural networks for the current (rollout phase) and previous (proof-of-concept phase) AI tools.

Class segmented	Mean IoU (proof-of-concept phase)	Mean IoU (rollout phase)
Background	78.8%	85.6%
All cell types	71.1%	79.0%
Neutrophils and eosinophils	57.5%	61.5%
Plasmocytes	55.2%	58.7%
Lymphocytes	52.5%	56.1%
Eosinophils	61.0%	63.2%
Overall average	63%	67%

Abbreviations: AI, artificial intelligence; IoU, Intersection over Union.

**Table 3.** Confusion matrix: correlations between Nancy Index score assigned by histopathologists (true label) and Nancy Index score assigned by AI (predicted label) for (A) current (rollout phase) and (B) previous (proof-of-concept phase) AI tools.

		Predicted label					
	Nancy Index	0	1	2	3	4	Number of images
(A) Current AI tool							
True label	0	0.8	0.16	0.04	0	0	25
	1	0.4	0.4	0.1	0.1	0	10
	2	0	0	0.75	0.25	0	8
	3	0	0	0.18	0.73	0.09	22
	4	0	0	0	0.2	0.80	10
Total							75

(B) Previous AI tool							
True label	0	0.4	0	0.44	0.16	0	25
	1	0.1	0.2	0.4	0.3	0	10
	2	0	0	0.75	0.25	0	8
	3	0	0	0.23	0.73	0.04	22
	4	0	0	0	0.2	0.8	10
Total							75

Abbreviation: AI, artificial intelligence.

The ICC between histopathologists was 94.6% between histopathologists 1 and 2, 92.6% between histopathologists 2 and 3, and 89.2% between histopathologists 1 and 3. The average ICC was 92.1% among the histopathologists and 91.1% between the histopathologists and the AI histology tool.

As shown in Supplementary Figure S2, the rollout phase AI histology tool was highly correlated with the histopathologists’ assessment in the different stages of disease progression.

## Discussion

This multicenter study expands on a previous single-center proof-of-concept study^9^ to use deep learning AI techniques to assess histopathology slides from patients with UC and to categorize UC severity based on the Nancy Index. The previous proof-of-concept AI model predicted a Nancy Index score based on a 3-neural network structure to characterize images by cell and tissue type combination/location, and was trained on 200 images. This expanded model was trained on 791 images and predicts a Nancy Index score based on a 4-neural network structure to characterize images by cell and tissue type combination/location.

As shown by the IoU results, there was sufficient overlap between the segmentation neural networks and the type of cells annotated by the histopathologists, leading to an accurate quantification of the density of the cells and tissues of each type. The 4-neural networks in this rollout phase of the AI model were better able to identify the different cell and tissue types when compared with the proof-of-concept AI tool, which only used 3 segmentation neural networks. This led to the average accuracy being improved from 63% in the proof-of-concept model to 67% in the rollout model. This improvement means that there is a superior dataset on which the classification module can work.

The confusion matrix analysis demonstrated that the strongest correlation was at the extremes of the Nancy Index, with 80% correlation between predicted and true labels for Nancy Index scores of 0 or 4. The correlations were even stronger when 2 adjacent scores were combined, with 96% correlation for a true Nancy Index score of 0 being predicted as 0 or 1, and 100% correlation for a true Nancy score of 2 being predicted as 2 or 3. The confusion matrix analysis of the proof-of-concept model demonstrated a 40% correlation between predicted and true labels for the Nancy score.^9^ The confusion matrix analysis and the Nancy metrics for proof-of-concept and pilot current AI models confirm that model performance had improved, delivering higher accuracy while benefiting from the improved robustness associated with more training data.

This AI tool was found to be highly correlated with histopathologists’ assessments, with the average ICC of 92.1% among the histopathologists and 91.1% for AI vs histopathologists. The ICC between AI and histopathologists for the proof-of-concept AI tool was 87.2%.^9^ Thus, the ICC on unseen images has also improved in the new rollout phase AI model.

The high correlation of the performance of the AI method for measurement of histological disease activity in UC with histopathologists’ assessments suggests promising potential for clinical applications in IBD such as UC. Convolutional neural network models that segment tissues and classify cells across WSI colon biopsies have previously been used to develop tissue and cell models to generate human interpretable features or meta-features, which can then be used to predict Nancy Index scores.^10^ The models demonstrated that the human interpretable features had an excellent correlation with the consensus pathologists’ assessment of disease activity across the Nancy Index. Nancy scores predicted for slide images were similar to those given by subspeciality-trained pathologists.

Other studies have also used AI models to assess histopathology in UC biopsies using different histological grading methods. In one such study, a novel and simplified histological score, the PICaSSO Histologic Remission Index (PHRI), was developed to reflect microscopic mucosal inflammation and healing, and predict clinical outcomes and response to therapy. The PHRI was designed to be readily implemented into an AI model to detect histological remission.^11^ A convolutional neural network classifier was developed to detect neutrophils in WSIs and classify the sample as either histologic remission or nonremission based on the presence of neutrophils. The results showed that model sensitivity, specificity, and accuracy in predicting UC activity on the test set were 78%, 91%, and 86%, respectively. Another AI model has been developed and validated to evaluate UC biopsies using the PHRI, the Robarts Histopathology Index, and the Nancy Index.^6^

As with all machine learning applications, potential limitations are that AI models can only pick up the features that have been programmed; therefore, they may not pick up additional irregularities that are not incorporated in the Nancy score, such as dysplasia or cytomegalovirus infection. Inherent bias in the original choice of training samples may also be amplified or carried through as the tool advances and learns. This study, however, has been designed to mitigate such bias by including a broad range of histopathology samples from patients with varying degrees of disease activity, and processing those samples by more than 1 trained pathologist. The exclusion of atypical slides, while reasonable for a training dataset, is not reflective of real-world scenarios, so may limit generalizability of the tool under real-world conditions. Additionally, the AI model was trained to support automated assessment of images using Nancy score, and not specifically cell counts and classification. The intermediate Nancy indices (1, 2, and 3) are assigned based on the cell counts and density; however, the definition of low/high cell count and density in the Nancy Index system are not clearly specified. This presents classification problems for expert pathologists, and this issue is also faced by the AI model.

Development of AI-based histopathology technologies to aid clinical decision making is in relatively early stages. These novel technologies and implementations face challenges before broader acceptance and adoption, including the generalizability of findings, standardization between different tools, difficulties in understanding the precise nature of what a given AI method does, and the relative lack of clear guidance on what is required by regulatory authorities before wider adoption. As a relatively new technology, these issues are currently being explored and the field is developing rapidly.^12^

Further development and refinement of the AI histology tool we have described may lead to a valuable resource that can complement the work of histopathologists, enabling higher throughput and verification/validation of findings. A standardized and validated histological, AI-driven, automated scoring system for UC has great potential for use in daily practice to eliminate the subjectivity or lack of expertise of histopathologists, to improve efficiency in reading and interpretation, to reduce the need for training and resource use, and to assess the severity of disease activity for treatment decisions. Automated AI-based centrally read histology may also play a role in improving the accuracy of clinical trials and making the acquisition of data for histology end points quicker and less expensive. There is also the possibility that AI histology tools may be able to identify new histological features with a crucial role in UC disease activity that have not yet been detected by humans. Further work is ongoing to better understand and explore the full implications of the use of this AI tool for histopathologic assessment and classification in IBD.

## Supplementary Data

Supplementary data is available at *Inflammatory Bowel Diseases* online.

izae204_suppl_Supplementary_Figures_S1-S2

## Data Availability

The datasets, including the redacted study protocol, redacted statistical analysis plan, and individual participants’ data supporting the results reported in this article, will be made available within 3 months from initial request to researchers who provide a methodologically sound proposal. The data will be provided after its de-identification, in compliance with applicable privacy laws, data protection, and requirements for consent and anonymization.

## References

[1] Ordás I , Eckmann L, Talamini M, Baumgart DC, Sandborn WJ. Ulcerative colitis. Lancet. 2012;380(9853):1606-1619. doi: https://doi.org/10.1016/S0140-6736(12)60150-0 22914296

[2] Raine T , Bonovas S, Burisch J, et al ECCO guidelines on therapeutics in ulcerative colitis: medical treatment. J Crohns Colitis. 2022;16(1):2-17. doi: https://doi.org/10.1093/ecco-jcc/jjab178 34635919

[3] Turner D , Ricciuto A, Lewis A, et al; International Organization for the Study of IBD. STRIDE-II: an update on the Selecting Therapeutic Targets in Inflammatory Bowel Disease (STRIDE) Initiative of the International Organization for the Study of IBD (IOIBD): determining therapeutic goals for treat-to-target strategies in IBD. Gastroenterology. 2021;160(5):1570-1583. doi: https://doi.org/10.1053/j.gastro.2020.12.031 33359090

[4] Bryant RV , Burger DC, Delo J, et al Beyond endoscopic mucosal healing in UC: histological remission better predicts corticosteroid use and hospitalisation over 6 years of follow-up. Gut. 2016;65(3):408-414. doi: https://doi.org/10.1136/gutjnl-2015-309598 25986946

[5] Colman RJ , Rubin DT. Histological inflammation increases the risk of colorectal neoplasia in ulcerative colitis: a systematic review. Intest Res. 2016;14(3):202-210. doi: https://doi.org/10.5217/ir.2016.14.3.202 27433141 PMC4945523

[6] Iacucci M , Parigi TL, Del Amor R, et al Artificial intelligence enabled histological prediction of remission or activity and clinical outcomes in ulcerative colitis. Gastroenterology. 2023;164(7):1180-1188.e2. doi: https://doi.org/10.1053/j.gastro.2023.02.031 36871598

[7] Le HD , Pflaum T, Labrenz J, et al Interobserver reliability of the Nancy Index for ulcerative colitis: an assessment of the practicability and ease of use in a single-centre real-world setting. J Crohns Colitis. 2023;17(3):389-395. doi: https://doi.org/10.1093/ecco-jcc/jjac146 36282973

[8] Stidham RW , Takenaka K. Artificial intelligence for disease assessment in inflammatory bowel disease: how will it change our practice? Gastroenterology. 2022;162(5):1493-1506. doi: https://doi.org/10.1053/j.gastro.2021.12.238 34995537 PMC8997186

[9] Peyrin-Biroulet L , Adsul S, Dehmeshki J, Kubassova O. DOP58 An artificial intelligence-driven scoring system to measure histological disease activity in ulcerative colitis. J Crohns Colitis. 2022;16(Supplement_1):i105. doi: https://doi.org/10.1093/ecco-jcc/jjab232.097 PMC11485311 38590110

[10] Najdawi F , Sucipto K, Mistry P, et al Artificial intelligence enables quantitative assessment of ulcerative colitis histology. Mod Pathol. 2023;36(6):100124. doi: https://doi.org/10.1016/j.modpat.2023.100124 36841434

[11] Gui X , Bazarova A, Del Amor R, et al PICaSSO Histologic Remission Index (PHRI) in ulcerative colitis: development of a novel simplified histological score for monitoring mucosal healing and predicting clinical outcomes and its applicability in an artificial intelligence system. Gut. 2022;71(5):889-898. doi: https://doi.org/10.1136/gutjnl-2021-326376 35173041 PMC8995819

[12] Kim I , Kang K, Song Y, Kim T-J. Application of artificial intelligence in pathology: trends and challenges. Diagnostics (Basel). 2022;12(11):2794. doi: https://doi.org/10.3390/diagnostics12112794 36428854 PMC9688959

