# Supplementary material for: Deployment of an Artificial Intelligence Histology Tool to Aid Qualitative Assessment of Histopathology Using the Nancy Histopathology Index in Ulcerative Colitis
Source: Inflamm Bowel Dis. 2024 Sep 16;31(6):1630–6. doi: 10.1093/ibd/izae204 (PMC12166296; doi:10.1093/ibd/izae204)

# Supplementary Data

**Supplementary Figure S1**. Segmentation by the segmentation neural networks.

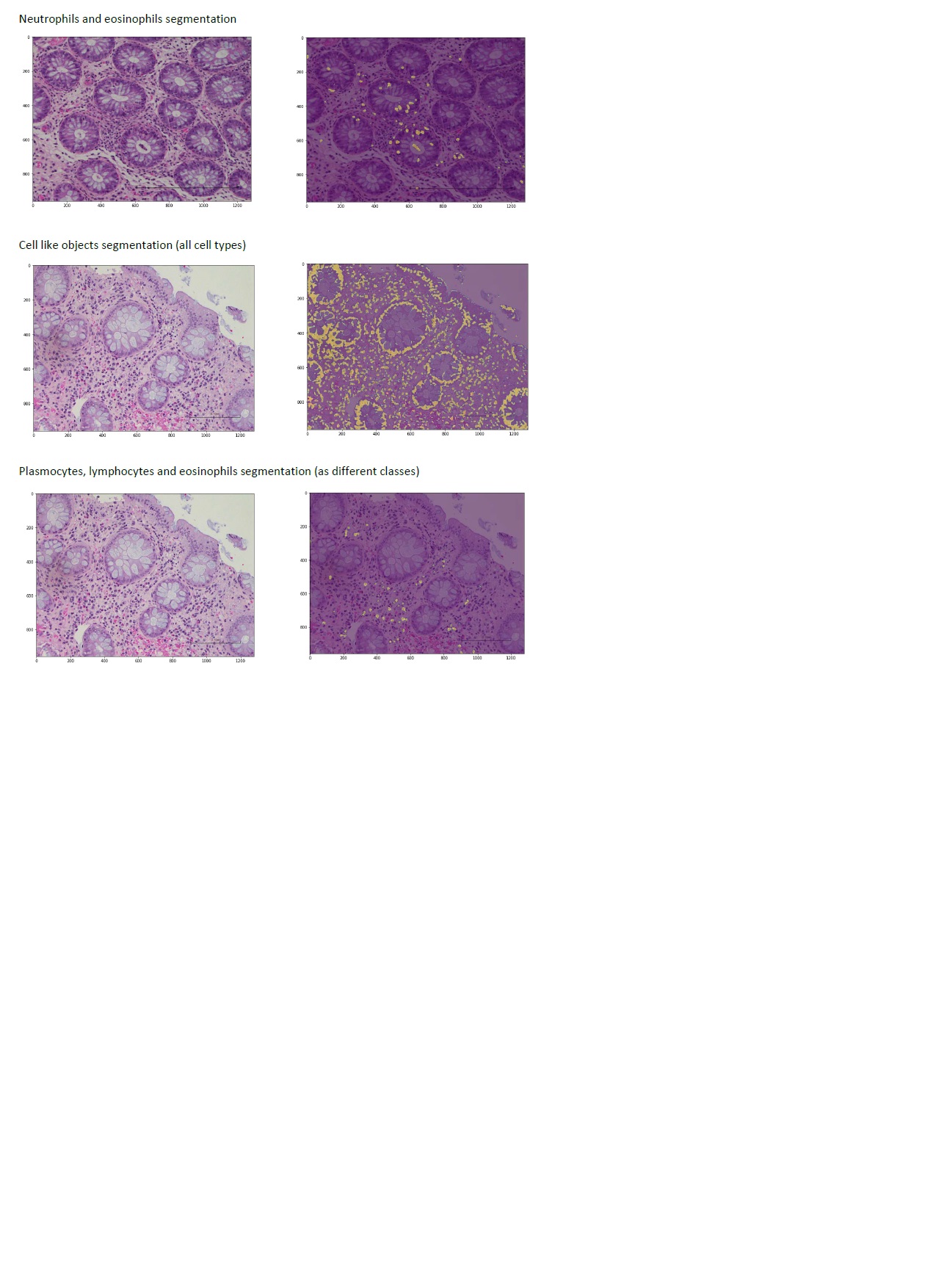


**Supplementary Figure S2.** Accuracy of the AI histology tool across stages of disease progression.


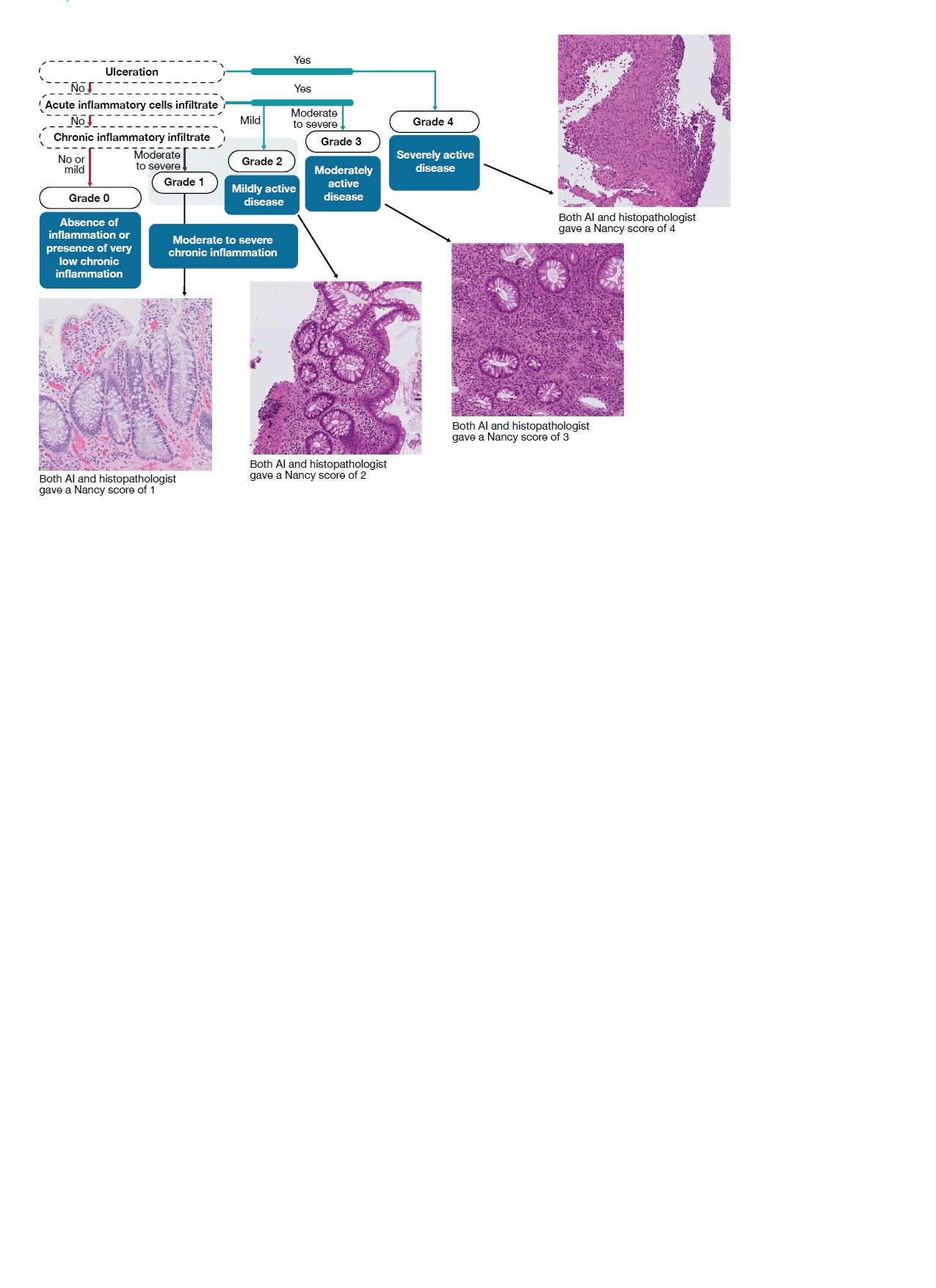

Supplement: izae204_suppl_Supplementary_Figures_S1-S2 [file izae204_suppl_supplementary_figures_s1-s2.docx]
